# Reduction in ATP Levels Triggers Immunoproteasome Activation by the 11S (PA28) Regulator during Early Antiviral Response Mediated by IFNβ in Mouse Pancreatic β-Cells

**DOI:** 10.1371/journal.pone.0052408

**Published:** 2013-02-01

**Authors:** Wieke Freudenburg, Madhav Gautam, Pradipta Chakraborty, Jared James, Jennifer Richards, Alison S. Salvatori, Aaron Baldwin, Jill Schriewer, R. Mark L Buller, John A. Corbett, Dorota Skowyra

**Affiliations:** 1 Edward A. Doisy Department of Biochemistry and Molecular Biology, Saint Louis University School of Medicine, Saint Louis, Missouri, United States of America; 2 Department of Molecular Microbiology and Immunology, Saint Louis University School of Medicine, Saint Louis, Missouri, United States of America; 3 Department of Biochemistry, Medical College of Wisconsin, Milwaukee, Wisconsin, United States of America; National Institutes of Health, United States of America

## Abstract

Autoimmune destruction of insulin producing pancreatic β-cells is the hallmark of type I diabetes. One of the key molecules implicated in the disease onset is the immunoproteasome, a protease with multiple proteolytic sites that collaborates with the constitutive 19S and the inducible 11S (PA28) activators to produce immunogenic peptides for presentation by MHC class I molecules. Despite its importance, little is known about the function and regulation of the immunoproteasome in pancreatic β-cells. Of special interest to immunoproteasome activation in β-cells are the effects of IFNβ, a type I IFN secreted by virus-infected cells and implicated in type I diabetes onset, compared to IFNγ, the classic immunoproteasome inducer secreted by cells of the immune system. By qPCR analysis, we show that mouse insulinoma MIN6 cells and mouse islets accumulate the immune proteolytic β1_i_, β2_i_ and β5_i_, and 11S mRNAs upon exposure to IFNβ or IFNγ. Higher concentrations of IFNβ than IFNγ are needed for similar expression, but in each case the expression is transient, with maximal mRNA accumulation in 12 hours, and depends primarily on Interferon Regulatory Factor 1. IFNs do not alter expression of regular proteasome genes, and in the time frame of IFNβ-mediated response, the immune and regular proteolytic subunits co-exist in the 20S particles. In cell extracts with ATP, these particles have normal peptidase activities and degrade polyubiquitinated proteins with rates typical of the regular proteasome, implicating normal regulation by the 19S activator. However, ATP depletion rapidly stimulates the catalytic rates in a manner consistent with levels of the 11S activator. These findings suggest that stochastic combination of regular and immune proteolytic subunits may increase the probability with which unique immunogenic peptides are produced in pancreatic β-cells exposed to IFNβ, but primarily in cells with reduced ATP levels that stimulate the 11S participation in immunoproteasome function.

## Introduction

T cell-mediated destruction of insulin producing pancreatic β-cells is the hallmark of type I diabetes, an autoimmune disease associated with various genetic and environmental factors. Viral infections have been recognized as one potential environmental trigger of onset, but the molecular basis of the link between antiviral defenses and autoimmunity is still unclear [1], [2]. In the currently dominating view, viral infection of pancreatic β-cells would induce a local immune response, accompanied by the production of cytokines that arrest viral replication and facilitate viral clearance, yet an undefined aspect of this inherently defensive process would also initiate the autoimmune response in genetically susceptible individuals. While the link between antiviral defenses and autoimmunity is unclear, central to this process is the family of interferons (IFNs) [3]. There are two major groups of interferons that are distinguished by several properties, including the timing of their induction and the cell type in which the induction takes place. Type I IFNs are produced shortly after viral infection and regulate the early steps of antiviral defense. This class includes IFNγ that is synthesized by infected cells in the absence of other cell types, and several types of IFNα that are synthesized primarily by leukocytes recruited to the infection site. In contrast, IFNγ, the only type II interferon, is synthesized by cells of the immune system upon their recruitment to infected cells, which takes place days or even weeks after infection.

While IFNγ is recognized as the primary inducer of innate and adaptive immunity in response to viral infections, a similar role has recently been attributed to type I IFNs. This conclusion was based on the observation that, in a chimpanzee model of acute HCV infection, the early antiviral responses mediated by type I IFNs correlate with an early activation of CD8+ T cells by a yet unknown antigen [4], [5]. Type I IFNs have long been implicated in the development of autoimmunity in type I diabetes in animal models and human patients [6]–[13], but little is known about the mechanisms by which this class of IFNs stimulates the immune response. Even if the mechanisms were fundamentally similar to those mediated by IFNγ, type I IFNs would activate the immune response during much earlier stages of viral replication and cellular antiviral response, thereby changing the context of the immune response activation.

A key aspect of immune response activation that could be altered most by the change of context is the production of antigenic peptides by the proteasome. This elaborate, multi-catalytic protease regulates many aspects of cell function and generates antigenic peptides for presentation by MHC class I molecules [14]–[17]. A large pool of antigenic peptides is derived from the robust, co-translational proteolysis of misfolded polypeptides, which eliminates about 30% of all new proteins produced in cells [18]. From this perspective, changes in gene expression could alter the pool of immunogenic peptides produced in cells. Under normal physiological conditions, this mechanism would play a minor role because only a small percent of total peptides generated by the proteasome is normally presented at the cell surface [19]–[21]. IFNs overcome this limitation by inducing the expression of several genes, including the genes that encode the three alternative proteolytic subunits: β1_i_ (also known as LMP2 or caspase-like site), β2_i_ (also known as MECL1 or trypsin-like site), and β5_i_ (also known as LMP7 or chymotrypsin-like site). These inducible subunits replace their regular counterparts within the proteasome and alter the pattern of protein cleavage to generate peptides that are more immunogenic [22]–[28]. Mice that lack all three of these subunits lack approximately 50% of the total epitopes presented by MHC class I molecules and reject wild-type cells [29], verifying that the inducible proteolytic subunits play a major role in antigen generation. However, it is unknown whether the complete replacement of proteolytic subunits is necessary to ensure an effective repertoire of immunogenic peptides, or whether a stochastic combination of regular and immune subunits could also generate unique immunogenic peptides that could trigger the early immune response.

Despite the differences in proteolytic subunits, the regular and immune proteasomes function in a similar manner. The proteolytic activity is always associated with the 20S core, which is composed of four tightly stacked heptameric rings of subunits, where the inner β-rings include proteolytic and non-proteolytic β subunits, while the outer α-rings form a gating channel. In each case, opening of the gating channel is necessary for substrate uptake and product release, and is facilitated by a dedicated activator. The 19S activator is expressed constitutively, recruits the majority of proteasomal substrates in a manner dependent on their polyubiquitination, and is composed of 19 distinct subunits, from which a ring of ATPases opens the 20S gate and unfolds substrates. The 11S activator (also called PA28) is composed of only two types of subunits [30], [31] that are typically expressed at low levels and that accumulate upon exposure to IFNγ or type I IFNs [5]. Because of its inducible nature, the 11S activator is thought to be associated primarily with the immunoproteasome, but it's role remains elusive. Unlike the 19S activator, the 11S activator does not require ATP and does not recruit polyubiquitinated substrates. It has been suggested that the 11S may promote ubiquitin-independent proteolysis [32] or may stimulate the release of peptides generated from substrates recruited by the 19S activator in the context of hybrid 11S/20S/19S particles [33], [34]. However, the mechanisms that coordinate the function of the two activators within such particles are unknown.

The possibility that the immunoproteasome is required for the activation of antigen presentation in pancreatic β-cells is suggested by several findings. IFNγactivates the expression of the MHC class I molecules together with the two intra-MHC encoded proteasomal subunits β1_i_ and β5_i_ in mouse β-cell lines TC3 and TC6-F7, and the corresponding activation of antigen presentation is attenuated by the proteasome inhibitor MG132, thereby demonstrating dependence on proteasomal proteolysis [35]. The expression of β1_i_ and β5_i_ is similarly stimulated by IFNγ in FACS-purified rat β-cells [36] and in a rat β-cell line [37]. However, no previous study has actually characterized the assembly, function, and regulation of the immunoproteasome in pancreatic β-cells exposed to either IFNγ or type I IFNs.

To address this issue, we analyzed the effect of IFNβ on the expression, assembly, and function of the immunoproteasome and the 11S activator in mouse islets, and in the mouse insulinoma MIN6 cells that can be grown in homogeneous *in vitro* cultures and yet retain many key aspects of pancreatic β-cell function. We report that both MIN6 cells and mouse islets accumulate the immunoproteasome and 11SmRNAs when exposed to IFNβor IFNγ. Higher concentrations of IFNβ than IFNγ are needed for similar expression, but in each case the expression is transient, with maximal mRNA accumulation in 12 hours, and depends primarily on Interferon Regulatory Factor 1 (IRF1). IFNs do not change the expression of regular proteasomal genes, and immuno-precipitation and quantitative Western blot analyses of cell extracts fractionated by size exclusion show that, in the time frame of the IFNβ mediated signaling, the immune and regular subunits co-exist in the same 20S particles. In the presence of ATP, these early immunoproteasome degrades model reporter peptides and polyubiquitinated proteins with rates typical of the regular proteasome, implicating normal function and regulation by the 19S activator. However, ATP depletion rapidly stimulates the proteolytic rates in a manner consistent with accumulation of the 11S activator. These data suggest that stochastic combination of regular and immune proteolytic subunits increases the probability with which unique immunogenic peptides are generated from proteasomal substrates, but primarily in cells with reduced ATP levels that promote 11S participation in immunoproteasome function.

## Materials and Methods

### Ethics Statement

The Animal Care and Use Committees at Saint Louis University and at the Medical College of Wisconsin have approved all animal studies in this report. Five mice were group housed in micro-isolator cages within the barrier facilities at Saint Louis University and MCW Biomedical Resource Center. Animals were maintained on a cycle of 12 hours of light and 12 hours of dark, with lights on at 6∶00 am. Mice were provided with water and standard chow ad libitum, and were not treated prior to being euthanized. Mice were euthanized using CO_2_ followed by cervical dislocation immediately prior to isolation of islets.

### Animals, cell lines, and cell culture materials

C57BL/6 mice were purchased from Harlan (Indianapolis, IN). IRF1^−/−^ mice on a C57BL/6 background and wild-type C57BL/6 controls were purchased from The Jackson Laboratory (Bar Harbor, ME). MIN6 cells, DMEM medium with 10% heat-inactivated fetal calf serum and 1x L-glutamine and CMRL-1066 medium with 10% heat-inactivated fetal calf serum, 1x L-glutamine, 100 units/ml penicillin and 100 mg/ml streptomycin were obtained from Washington University Tissue Culture Support Center (St. Louis, MO). Mouse IFNα and IFNβ were obtained from PBL Interferon Source (Piscataway, NJ). Mouse IFNγ was obtained from R&D Systems (Minneapolis, MN).

### Islet isolation and in vitro treatment

Islets were isolated from C57BL/6J wild type or IRF1^−/−^ mice by collagenase digestion [38], plated at concentration of 150 islets/0.4 ml in a 19 mm well and cultured overnight in CMRL-1066 medium before treatment with interferons.

### Culture of MIN6 cells

MIN6 cells were harvested by treatment with 0.05% trypsin, 0.02% EDTA at 37°C, washed with DMEM, replated at 50–70% confluency (2×10^5^/0.4 ml in a 19 mm well), and cultured overnight prior to further treatment with interferons.

### Primers for qPCR

See Table 1. The primers were designed to exons of specific genes in a manner that include (procedure A) or does not include (procedure B) sequence of the adjacent intron that only in case of genomic DNA contamination would promote synthesis of a larger product. PCR primers for GAPDH [39], IRF1 [40] and IRF3 [41] have been described previously. All primers were purchased from Integrated DNA Technologies (Coralville, IA).

**Table 1:** Primer sequences used in this study.

Primers Procedure A (forward and reverse)
β1_i_: 5′-ATGGCAGTGGAGTTTGACGG-3′; 5′-ATACCTGTCCCCCCTCACATTG-3′
β2_i_: 5′- GCTTGTGTTCCGAGATGGAGTC-3′; 5′-TCCGTTCAAATCAACCCCG-3′
β5_i_: 5′-CATTCCTGAGGTCCTTTGGTGG-3′; 5′-ATGCGTTCCCCATTCCGAAG-3′
β2: 5′-CGCAAAAAAGGGGTTCAAGC-3′; 5′-AGATGCTGTAGAGATGAGGTCCAG-3′
Psmd4: 5′-TCCTATTCTGGCTGGTGAAGGC-3′; 5′-CAAACTCCTGCTGGTTGATGGTC-3′
Psmd8: 5′-GCCTCAATCTCCTCTTCCTGCTATC-3′; 5′-GTCTGTCATCTTTTTGGGTGTGC-3′
11Sα: 5′-GTCAAAGAGAAAGAGAAGGAGGAGC-3′; 5′-GGTGTGAAGGTTGGTCATCAG C-3′
11Sβ: 5′-AAGTCCCTAAGTGTGGCTACCTCC-3′; 5′-GGCTTTTCTTCACCCTTCGG-3′
Actin: 5′-AGCCATGTACGTAGCCATCCAGGCTG-3′; 5′-TGGGTACATGGTGGTACCACCAGACA-3′

Primers procedure B (forward and reverse)
β1_i_: 5′-ACAGCCCTTTACCATCGGCGG-3′; 5′-CCGGCACTCCTCAGGGGTCA-3′
β2_i_: 5′-GGGGCTGGAGTAGCTGCGGA-3′; 5′-GCGCAGGATACGGGTGACCG-3′
β5_i_: 5′-CCACTGGCAGCGGGAACACC-3′; 5′-AGCTCTGCGGCCAAGGTCGT-3′
β1: 5′-CAGTCCTTTGCCATCGGAGGCT-3′; 5′-CGTCCTTGGTCATGCCTTCCC G-3′
β2: 5′-ATCAAGGTTACATTGGTGCAGCCC-3′; 5′-ATGCTGTAGAGATGAGGTCCAGTAACA-3′
β5: 5′-GCCTATGATCTGGCCCGCCG-3′; 5′-CCAGCCATCCTCCCGCACG-3′
HPRT: 5′-GGCCATCACATTGTGGCCCT-3′; 5′-TGTAATCCAGCAGGTCAGCAAAGAAC-3′

### qPCR

Total RNA was isolated using the RNeasy kit (Qiagen, Valencia, CA), without (procedure A) or with (procedure B) DNase I treatment (Epicentre, Madison, WI). cDNA was synthesized using oligo(dT) and the reverse transcriptase Superscript preamplification system (Invitrogen, Carlsbad, CA; procedure A), or random hexamer priming with the high capacity cDNA Reverse Transcription Kit (Applied Biosystems, Foster City, CA; procedure B). Real-time PCR was performed using Power SYBR PCR Mastermix (Applied Biosystems, Foster City, CA) and a Research DNA Engine Opticon II thermo cycler with continuous fluorescence detection (MJ Research, Waltham, MA). The synthesis of a single product was verified by agarose gel electrophoresis and the mRNA levels were normalized to the constitutively expressed GAPDH mRNA (procedure A) or hypoxanthine-guanine phosphoribosyltransferase (HPRT) mRNA (procedure B). In procedure A, mRNA levels of target genes in interferon treated samples were calculated as fold change compared to the same target gene in untreated samples. In procedure B, mRNA levels were calculated adjusting for amplification efficiency of each individual primer set [42] and normalized to control reactions with genomic DNA, thereby allowing direct comparison between expression of different genes. Genomic DNA was obtained from MIN6 cell extracts using QIAamp DNA extraction kit (Qiagen, Valencia, CA). Standard errors of the mean were calculated using three independent experiments.

### Antibodies

Abcam Inc. (Cambridge, MA): rabbit antibodies specific to human β1_i_/LMP2 (ab3328), mouse β5/PSMB5/X (ab3330), human α5 (ab11437), and human β5_i_/LMP7 (ab3329); Enzo Life Sciences (Plymouth Meeting, PA): rabbit antibodies specific to mouse β5_i_/LMP7 (BML-PW8200), mouse 11Sα/PA28α (BML-PW8185), mouse 11Sβ/PA28β (BML-PW8240), and dog Calnexin (ADI-SPA-860); mouse antibodies specific to prosbox1 motif common to alpha1-7 subunits of the 20S core (BML-PW8195), human Rpt1 (BML-PW8825), human Rpt4 (BML-PW8830), and human Rpt5 (BML-PW8770); Santa Cruz (Santa Cruz, CA): rabbit antibodies specific to IRF1 (sc-640); mouse antibodies specific to human GAPDH (sc-59541) and acetylated sea urchin α tubulin (sc-23950); SIGMA (St. Louis, MO): mouse antibodies specific to poly-histidine tag (H1029), and rabbit antibodies specific to bovine ubiquitin (U5379); rabbit antibodies specific to histone H3 (Upstate, NY); rabbit antibodies specific to mouse Sal1 [43]; horseradish peroxidase-conjugated donkey anti-rabbit and anti-mouse antibodies from Promega (Fitchburg, WI) and Jackson ImmunoResearch (West Grove, PA).

### Recombinant proteins

Mouse recombinant β5 and β5_i_ proteins were expressed in *E. coli* from the cDNAs MGC-19313 (β5) and MGC-6535 (β5_i_) purchased from ATCC (Manassas, VA) and subcloned into the pET15b vector that allows an expression of N-terminally tagged His6 proteins in *E. coli* (this work). Briefly, the cDNAs were amplified by PCR using the following oligonucleotides: 5′ β5 XhoI pU50 5′-ccg CTCGAG cg ATG GCG CTG GCT AGC GTG TTG-3′, 3′ β5 NotI pU50 5′-atagttta GCGGCCGC c TCA GGG GAC AGA TAC ACT-3′, 5′ β5_i_ Xho pU50 5′-ccg CTCGAG cg ATG GCG TTA CTG GAT CTG TGC-3′, and 3′ β5_i_ BamHI pU50 5′-cg GGATCC g TCA CAG AGC GGC CTC TCC GTA-3′. The PCR products were subcloned into pUni50, one of the Cre-Lox-based univector plasmid-fusion systems [44], sequenced and recombined with the Cre-Lox compatible final expression vector pET15b. The recombinant His-β5 and His-β5_i_ proteins were expressed in BL21(DE3) lysS cells and purified using Nickel agarose (Sigma-Aldrich, St. Louis, MO) accordingly to the manufacturer's instructions.

### Preparation of whole cell extracts for Western blot using SDS-sample buffer

2×10^5^ MIN6 cells or 150 mouse islets were lysed directly in cell culture wells with 35 µl of preheated SDS-loading buffer. This type of extract contained only unfolded proteins and was used only for Western blot analysis, typically using 30% of the total sample per single analysis.

### Preparation of whole cell extracts for analysis of the 20S assembly using extraction buffer with Triton X-100

MIN6 cells (5×10^6^) treated with IFNs as indicated were washed with PBS in culture wells, lysed using 7 ml of ice-cold extraction buffer A (50 mM Hepes pH 7, 200 mM KCl, 0.5 mM DTT, 10 mM MgCl_2_, 2 mM ATP, 0.5% Triton-X, and 5% glycerol), blast frozen and stored at −80°C. Prior to analysis, lysates were clarified by centrifugation at 12,000 g at 4°C for 25 minutes followed by 10-fold concentration on Centricon-50 units (Millipore, Billerica, MA). This type of extract was used for HPLC and immuno-precipitation of the 20S particles, but due to presence of Triton X-100 could not be reliably used for functional assays.

### Preparation of detergent-free, low salt extracts for analysis of the 19S and 11S-dependent proteolytic rate of the 20S particles

Large-scale cultures of MIN6 cells (10^8^ cells) or mouse islets (1500 islets) treated with IFNs as indicated were washed with ice cold PBS directly in culture flasks followed by removal of the attached cells by scraping in PBS, concentration of the removed cells by centrifugation, and blast freezing of the wet cell pellets in liquid nitrogen. Cell pellets were thawed and extracted by five sequential, 2 minute each, incubations with two pellet volumes of an ice-cold extraction buffer B (50 mM Tris, pH 7.2, 50 mM KCl, 5 mM MgCl_2_, 2 mM ATP, and 0.5 mM DTT). The first two sequential extracts that contained most of the total cellular proteasomes (>95%) and cytosolic markers were combined as a detergent-free extract (∼2 mg/ml protein), aliquoted, and stored at −80C.

### Western blot analysis

Protein extracts, prepared as indicated, were separated by 10% sodium dodecylsulfate-polyacrylamide gel electrophoresis (SDS-PAGE) and transferred to nitrocellulose (GE Healthcare, Piscataway, NJ) by 16-hour electrotransfer at 30 V, or to PVDF membrane (Pall Life Sciences, Pensacola, FL) by 1 hour semidry transfer at 60 mA. The membranes were blocked with fat-free bovine milk solution and incubated for 1 hour at room temperature with the indicated antibody diluted 1∶1000, or as indicated, in immunoblotting buffer (10 mM Tris, pH 8.0, 150 mM NaCl, and 0.05% Tween 20). Horseradish peroxidase-conjugated donkey anti-mouse and anti-rabbit secondary antibodies were used at 1∶25,000 dilution (Promega) or 1∶5000 dilution (Jackson ImmunoResearch). ECL detection was performed according to the manufacturer's specifications (PerkinElmer, Waltham, MA).

### HPLC

1 mg of whole cell MIN6 protein extract with 0.5% Triton X-100 was loaded in a volume of 0.5 ml on Superdex 200 10/300 GL column (GE Healthcare, Piscataway, NJ) pre-equilibrated with HPLC running buffer (50 mM Hepes pH7, 200 mM KCl, 0.5 mM DTT, 10 mM MgCl_2_, and 0.25 mM ATP) and separated by HPLC (Waters, Milford, MA) at 4^o^C with a flow rate of 0.5 ml/min and fraction size 0.5 ml. 5% (25 μl) of gel filtration (GF) fractions 5–29 were analyzed by SDS-PAGE and Western blot, as indicated. The Superdex column was calibrated with gel filtration standards from BioRad (Hercules, CA).

### Immuno-precipitation

70 μg of the same MIN6 cell extract that was analyzed by HPLC (see above) were diluted to 240 μl, supplemented with 50 μl of IgG (1 μg/μl) specific to the indicated antigen (β5^C^: C-terminal peptide of β5 subunit; β5_i_^C^: C-terminal peptide of β5_i_ subunit; β5_i_^M^: middle part of subunit β5_i_, amino acids 23–223), and control IgG from pre-immune rabbit) and 10 μl of Protein A beads. After 1 hour of tumbling at 4°C the beads were collected by low speed centrifugation, washed with TBST buffer (3×1 ml), suspended in 2x Sample loading buffer, boiled and analyzed by SDS-PAGE/Western blot, as indicated.

### Analysis of the ATP-dependent and ATP-independent chymotrypsin-like (CTL) activity of the 20S proteolytic core

200 μl of 0.2 mM Suc-LLVY-AMC substrate prepared from 10 mM stock in DMSO and the indicated reaction buffer (50 mM Tris pH 7.2, 50 mM KCl, 5 mM MgCl_2_, 0.1 mM EDTA, 0.1 mg/ml BSA and either 1 mM ATP or no ATP) was preheated in a 37°C water bath for 2 minutes and transferred to a semimicro quartz cuvette placed in a temperature controlled holder in a Varian Fluorescence Spectrophotometer. 10 μl of the indicated protein extract with 1 mM ATP adjusted to 1mg/ml concentration was diluted in a separate tube to 200 μl of the reaction buffer (with no extra additions, with the indicated proteasome inhibitor, and/or with 2 units of apyrase, as indicated), followed by incubation at 37°C for 2 minutes, and addition of the protein mixture to the Suc-LLVY-AMC substrate. The appearance of AMC fluorescence was monitored in real time every 0.2 second for 60 minutes, with excitation at 380 nm and emission 460 nm. The reaction rate per minute was calculated using the Varian Eclipse software.

## Results

### IFNβ induces accumulation of immunoproteasome and 11S mRNAs in a manner preceeded by activation of IRF1 gene expression and prevented by IRF1 gene knockout

**Figure 1:**
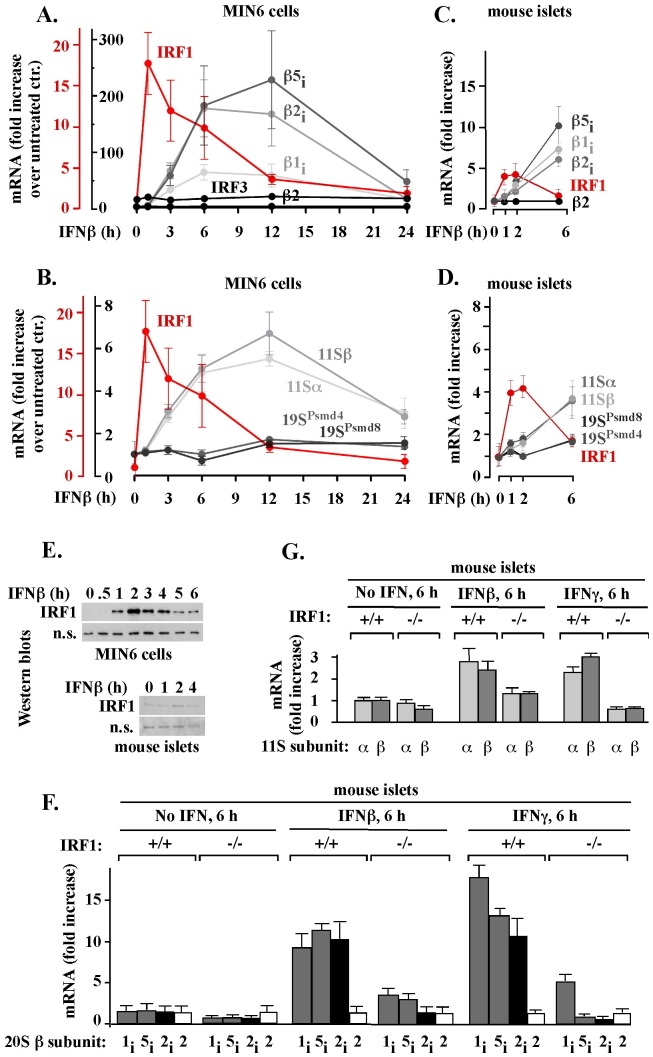
IFNβ stimulates accumulation of immunoproteasome and the 11S mRNAs in a manner preceded by IRF1 gene expression and prevented by IRF1 gene knockout. Fold increase in the indicated mRNA levels was measured by qPCR (procedure A) in MIN6 cells (A, B) or mice islets (C, D, F, G) exposed to 150 unit/ml of IFNβ or IFNγ. The results represent the mean ± S.E.M. of three (MIN6) or five (islets) experiments. (E) IRF1 protein was visualized by Western blot in whole-cell extracts from MIN6 cells (top) and mouse islets (bottom) treated with IFNβ or IFNγ, as described above; a non-specific (n.s.) protein band serves as a loading control.

Little is known about the function and regulation of the immunoproteasome in pancreatic β-cells, especially in response to IFNβ, a type I IFN secreted by virus-infected cells and implicated in type I diabetes onset. To test how IFNβ affects expression of immunoproteasome and 11S genes in pancreatic β-cells, we first used mouse insulinoma MIN6 cells that can be grown in homogeneous *in vitro* cultures. Since immunoproteasome function and regulation has not been previously characterized in pancreatic β-cell lines, the initial choice of MIN6 cells was based on the observation that this cell line recapitulates other key features of pancreatic β-cell function [45]. In response to 150 units/ml of IFNβ, the amounts of immunoproteasome β1_i_, β2_i_ and β5_i_ mRNAs increased 70–180 fold within 6 hours and 80–220 fold within 12 hours, followed by a decline to uninduced levels in approximately 24 hours (Fig. 1A, β1_i_, β2_i_ and β5_i_). Under the same conditions, the amount of the representative constitutive proteasomal β2 mRNA did not change, verifying the selectivity of IFNβ-mediated changes (Fig. 1A, β2). IFNβ also stimulated an increase in the levels of mRNAs that encode α and β subunits of the inducible 11S activator, with a maximum, 6-fold, increase in 12 hours and a decline to nearly normal expression in 24 hours (Fig. 1B, 11Sα and 11Sβ). Under the same conditions, mRNA levels of the representative Psmd4 and Psmd8 subunits of the constitutive 19S activator were increased no more than 1.5-fold (Fig. 1B, 19S^Psmd4^ and 19S^Psmd8^).

To confirm that primary tissue responds in a manner similar to MIN6 cells, we tested the effect of IFNβ on immunoproteasome and 11S mRNA levels in isolated mouse islets, in which β-cells account for 65–80% of the total cells. Consistent with the results obtained with MIN6 cells, IFNβ stimulated by 5 to 10-fold the accumulation of immunoproteasome β1_i_, β2_i_ and β5_i_ mRNAs in 6 hours of treatment, with no effect on the constitutive β2 (Fig. 1C), β3, β5, and α4 mRNAs (data not shown). Similarly, IFNβ stimulated the accumulation of αand β 11S mRNAs by 4-fold after 6 hours, whereas, under the same conditions, the accumulation of the representative 19S mRNAs Psmd4 and Psmd8 was stimulated by only 1.5 fold (Fig. 1D). Thus, IFNβ induced expression of immunoproteasome and 11S genes in isolated mouse islets.

To get an insight into the regulation of immunoproteasome and 11S genes in pancreatic β-cells, we tested a role of IRF1, the inducible transcription factor that stimulates expression of immunoproteasome genes in many other cell types exposed to IFNγ [46]–[51]. The IFNβ-mediated accumulation of immunoproteasome and 11S mRNAs in MIN6 cells (Fig. 1A–B, gray lines) and mouse islets (Fig. 1C–D, gray lines) was preceded by accumulation of IRF1 mRNA, with a maximum in 1–2 hours of treatment (Fig. 1A–D, red lanes). At least in MIN6 cells, mRNA level of IRF3, a non-inducible member of the IRF family, was unchanged (Fig. 1A), verifying the specificity of IRF1 gene activation. In agreement with IRF1 mRNA accumulation, IRF1 protein accumulated in MIN6 cells and mouse islets with a maximum in 2 hours (Fig. 1E). These results established a clear temporal relationship between the expression of IRF1, immunoproteasome, and 11S genes in pancreatic β-cells.

To directly test the role of IRF1 in the activation of immunoproteasome and 11S genes by IFNβ, we analyzed changes in immunoproteasome and 11S mRNA levels in islets isolated from wild type (IRF1^+/+^) and IRF1 knockout (IRF1^−/−^) mice. The lack of the IRF1 gene expression in the IRF1^−/−^ islets was confirmed by reverse transcriptase PCR (not shown). The islets were isolated from untreated mice and exposed to IFNβ for 6 hours *in vitro*. As a reference, a similar treatment was performed with IFNγ. The IRF1 gene knockout prevented accumulation of β2_i_ mRNA and severely compromised accumulation of β1_i_ and β5_i_ mRNAs (Fig. 1F). We observed similar effects with primers described by Namiki and colleagues [51] (data not shown). The IRF1 gene knockout also reduced the accumulation of α and β 11s mRNAs, except that this reduction was less severe in experiments with IFNβ than with IFNγ (Fig. 1G). This analysis showed that IFNγ or IFNβ induced expression of immunoproteasome and 11S genes in MIN6 cells and mouse islets in a manner dependent primarily on the inducible IRF1 transcription factor. However, additional factors may have also been involved, especially in the regulation of 11S genes by IFNβ.

### In the time frame of IFNβ-mediated responses, immune and regular proteolytic subunits are expressed concurrently, and in similar mRNA and protein levels

To understand the significance of the IFNβ-mediated activation of immunoproteasome gene expression, it was necessary to determine how this activation compares to the expression of regular proteasome genes, and to the activation of immunoproteasome gene expression by IFNγ.

**Figure 2:**
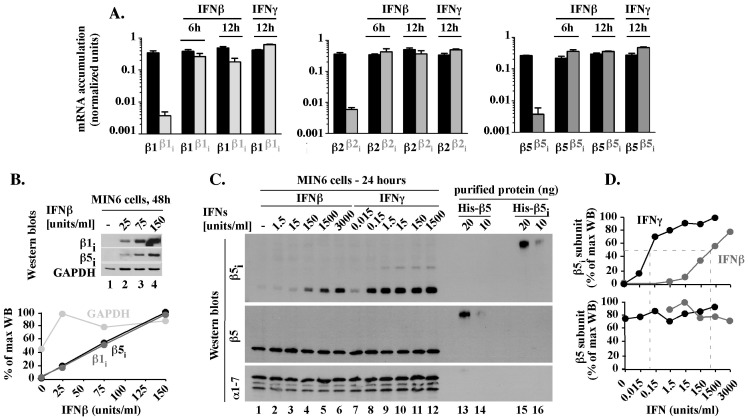
In MIN6 cells exposed to IFNβ, inducible and regular 20S mRNAs and proteins are expressed concurrently and in similar levels. (A). Comparison of inducible and regular 20S mRNA levels. mRNA levels of regular (black, β1, β2, β5) or immune (gray, β1_i_, β2_i_, β5_i_) subunits in MIN6 cells treated with 150 units/ml IFNβ or IFNγ for 6 or 12 hours were tested by qPCR, procedure B. Results are the mean ± S.E.M. of three experiments. (B). Accumulation of inducible 20S β1_i_ and β5_i_ proteins. Whole cell extracts from MIN6 cells treated for 48 hours with various concentrations of IFNβ were analyzed by Western blot with antibodies specific to β5_i_ and β1_i_ subunits. GAPDH is shown as a loading control. Quantitation of the WB data is shown as a percentage of the maximal accumulation for each analyzed protein. (C). Comparison of β5 and β5_i_ protein levels. MIN6 cells exposed for 24 hours to the indicated concentrations of IFNβ or IFNγ (lanes 1–12) were analyzed by Western blot next to 10 and 20 ng of mouse His-β5 and His-β5_i_ proteins expressed in, and purified from, *E. coli* (lanes 13–16). Levels of the 20S alpha subunits (α1-7 WB) are shown as loading control. (D). Quantitation of WB data presented in C. Protein levels represent a percentage of the maximal accumulation for each protein.

To compare the levels of different mRNAs, we performed qPCR analysis with primers specific to exons of immune β1_i_, β2_i_, and β5_i_ and regular β1, β2, and β5 genes, using cDNA and genomic DNA as templates (qPCR procedure B). The results were adjusted for amplification efficiency of each individual primer set and normalized to control reactions with genomic DNA, thereby allowing direct comparison between expression of different genes. In untreated MIN6 cells, immune β1_i_, β2_i_ and β5_i_ mRNAs levels were approximately 500-fold lower than constitutive β1, β2 and β5 mRNAs levels (Fig. 2A, compare gray and black bars in untreated cells). IFNs did not change the levels of constitutive β1, β2 and β5 mRNAs (Fig. 2A, all black bars). However, IFNs induced the accumulation of immune β1_i_, β2_i_, and β5_i_ mRNAs to levels that are similar to each other (Fig. 2A, compare gray bars + IFNβ, + IFNγ) and to constitutive β1, β2 and β5 mRNAs (Fig. 2A, compare gray and black bars + IFNβ, + IFNγ), with differences in the range of 2-fold. Thus, in the time frame of IFNβ-mediated responses, immune and regular proteasomal subunits were expressed concurrently and in similar mRNA levels.

The IFNβ-mediated accumulation of inducible β1_i_ and β5_i_ mRNAs did correlate with the accumulation of β1_i_ and β5_i_ proteins (Fig. 2B). To test how the protein levels of inducible and regular proteasomal subunits compare to each other, we calibrated the Western blot conditions in a manner ensuring similar sensitivities of detection of recombinant His-β5 and His-β5_i_ proteins (Fig. 2C, lanes 14–16). Using these conditions, we then analyzed the levels of β5 and β5_i_ proteins in MIN6 cells treated for 24 hours with various concentrations (0.015–3000 units/ml) of IFNβ or IFNγ. Each IFN induced an accumulation of β5_i_ protein to a similar level, but higher doses of IFNβ than IFNγ were required for similar accumulation, with 50% of the maximal β5_i_ protein levels observed with approximately 0.08 units/ml of IFNγ or 800 units/ml of IFNβ (Fig. 2C and 2D, β5_i_). Under the same conditions, levels of β5 protein were unchanged (Fig. 2C and D, β5). Thus, in MIN6 cells exposed for 24 hours to IFNβ or IFNγ, which follows the maximal level of inducible mRNAs by 12 hours and represents the time frame of IFNβ-mediated response, immune and regular subunits were expressed concurrently and at similar protein levels.

### Regular and immune proteolytic subunits expressed in MIN6 cells exposed to IFNβ co-exist in the same 20S core particles

**Figure 3:**
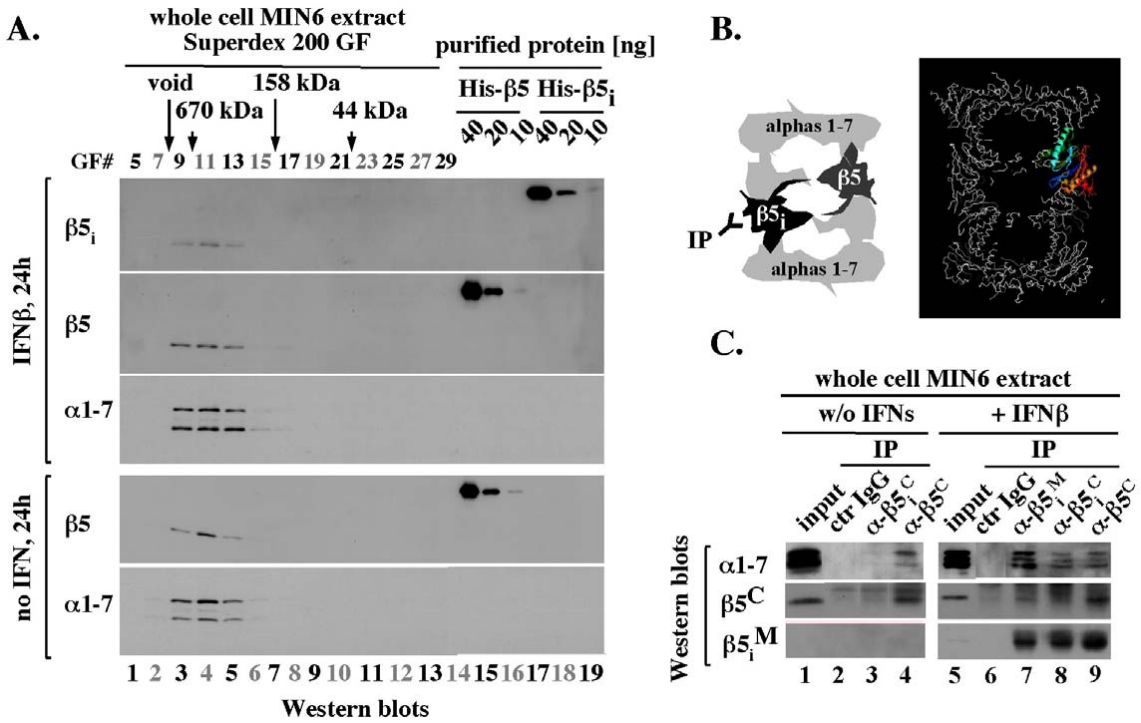
After 24 hours of treatment with IFNβ, inducible and regular subunits co-exist in the 20S particles. (A). Size exclusion chromatography. Size exclusion chromatography of extracts prepared from IFNβ treated (top 3 blots) and untreated (bottom 2 blots) MIN6 cells was performed as described in Methods and the indicated gel filtration fractions (GF# 5–29, lanes 1–13) were analyzed by Western blot next to the indicated amounts of purified mouse His-β5 and His-β5_i_ proteins (lanes 14–19). (B). Models with β5 and β5_i_ subunits in a single 20S particle (left), and with 20S structure in which one of the two β5 subunits is marked in color, with C-terminus in red (right). (C). Immuno-precipitation. MIN6 extracts analyzed in A were subjected to immuno-precipitation (IP) with the indicated antibodies or control (ctr.) IgG from a pre-immune rabbit. The immunoprecipitates were analyzed by Western blot, as indicated.

To test whether the inducible proteolytic subunits expressed in the presence of IFNβ are assembled in the 20S core particles, we performed size exclusion chromatography of extracts prepared from MIN6 cells treated for 24 hours with 150 units/ml of IFNβ. The inducible β5_i_ subunit eluted as an approximately 670 kDa complex (Fig. 3A, IFNβ, β5_i_, lanes 3–5). A similar elution profile characterized the constitutive 20S subunits β5 and alpha 1–7 in the presence and absence of IFNβ (Fig. 3A, IFNβ and “no IFN”, β5 and α1–7, lanes 3–5). We did not detect free β5 or β5_i_ subunits, which would elute as approximately 30 kDa proteins (Fig. 3A, gel filtration fractions 23–27, lines 10–12), even when we analyzed 10-fold larger samples (data not shown). The difference in β5 and β5_i_ protein levels in the high molecular weight complex (Fig. 3A, IFNβ, compare β5_i_ and β5 intensities in lanes 3–5) was consistent with the difference in the total β5 and β5_i_ protein levels observed before (Fig. 2C, β5 and β5_i_, lane 4). Thus, at least 90% of regular and immune subunits expressed in the presence of IFNβ were incorporated into the 20S particles.

IFNγ facilitates replacement of proteolytic subunits in the 20S particles, but complete replacement typical of mature immunoproteasomes may require several weeks, as it depends on removal of all regular subunits and pre-existing 20S cores, which have half-life of approximately 5 days [52], [53]. To test the possibility that during the early, IFNβ-mediated response, the regular and inducible subunits co-exist in the 20S particles we used an immuno-precipitation approach. Since each 20S core contains a duplicate of each βsubunit, a complex immuno-precipitated with antibodies specific to a regular subunit could contain an immune subunit and vice versa, but only if both versions are present in the same 20S particle (Fig. 3B, top, left). To address this possibility, we first identified antibodies that were subunit-specific under conditions of immuno-precipitation. We focused on antibodies specific to the C-terminal fragments of β5 and β5_i_ that would be located on the outside of the 20S structure (Fig. 3B, right, residues in red), and on antibodies generated against a large middle fragment of β5_i_ (β5_i_^M^, amino acids 23–230) that has several unique sequences located on the outside of the 20S core (data not shown). In experiments with extracts prepared from untreated MIN6 cells that expressed constitutive 20S cores only, alpha subunits (α1–7) were detectable in immuno-precipitates isolated with antibodies specific to the C-terminus of β5 subunit (β5^C^), but not β5_i_ subunit (β5_i_^C^) or control IgG (Fig. 3C, lanes 1–4). In contrast, in extracts from IFNβ-treated MIN6 cells, alpha and β5_i_ subunits were detected in complexes immuno-purified via both β5_i_ (Fig. 3C, lanes 7, 8) and β5 (Fig. 3C, lane 9). These results showed that regular and inducible subunits expressed in MIN6 cells co-existed in the same 20S particles.

### Functional analysis of detergent-free MIN6 cell extracts suggests that reduction in ATP levels triggers participation of the 11S activator in immunoproteasome function

To perform functional analysis of immunoproteasome complexes assembled in MIN6 cells exposed to IFNβ and IFNγ, we sought to develop a cell extraction strategy that will be most likely to preserve authentic interactions between the 20S, 19S and 11S particles. Such a strategy should avoid the use of high salt concentrations, detergents, and long incubation times that could cause changes in protein-protein interactions and/or posttranslational modifications.

**Figure 4:**
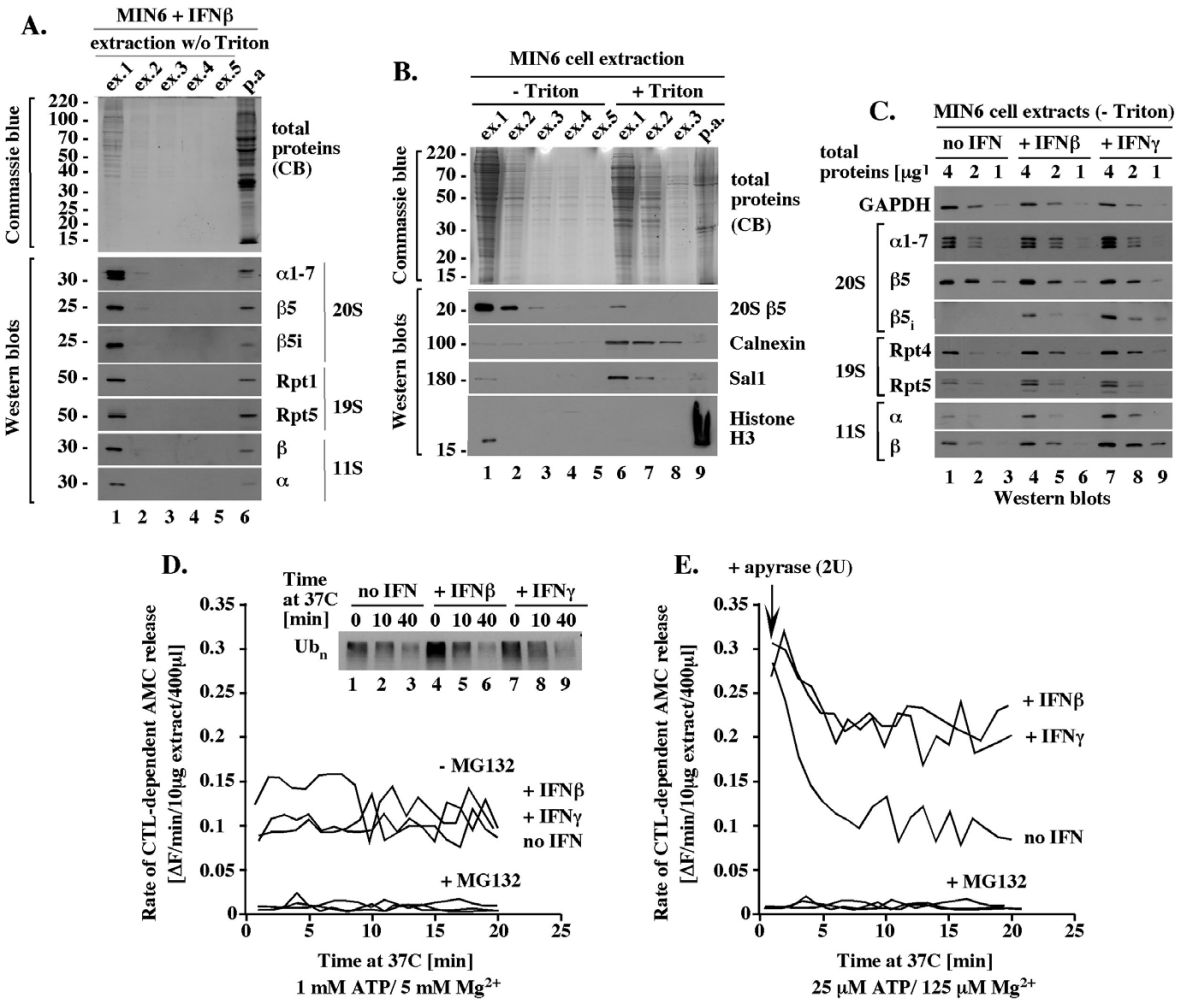
Functional analysis of detergent-free MIN6 cell extracts suggests that reduction in ATP levels triggers participation of the 11S in the 20S function. (A). Rapid extraction of proteasome components with a hypotonic, detergent-free buffer. Pellet of 3×10^6^ MIN6 cells treated with 300 U/ml of IFNβ for 24 hours was subjected to 5 sequential incubations with 2 pellet volumes (40 μl) of a hypotonic, ATP-rich, extraction buffer without detergent (ex. 1–5, -Triton; Methods) followed by solubilization of all proteins in the remaining pellet (pellet after: p.a.) by boiling with SDS-sample buffer (Methods). 12.5% of each extract was separated by SDS-PAGE and analyzed by Western blot or Commassie blue stain, as indicated. (B). The detergent-free 20S complexes are extracted together with cytosolic proteins. Experiment like in A except that the 5 sequential incubations with a detergent-free buffer (ex. 1–5, -Triton) were followed by additional 3 sequential extractions with the same buffer supplemented with 0.5% Triton (ex. 1–3,+0.5% Triton, Methods) prior to extraction of the remaining pellet with SDS sample buffer. (C). Quantitative Western blot analysis of the 20S, 19S and 11S components in detergent-free MIN6 cell extracts. Equal amounts of the total proteins (4, 2, and 1 μg) from the indicated detergent-free extracts were analyzed by Western blot with antibodies specific to the indicated proteins. (D). 20S activity in ATP-rich, detergent-free MIN6 extracts. The indicated detergent-free cell extracts with 1 mM ATP were tested *in vitro* for chymotrypsin-like (CTL) peptidase activity in the presence or absence of 5 μM MG132 (graph) and for degradation of polyubiquitinated proteins (Western blot insert) as described in Methods. (E). 20S activity in detergent-free MIN6 extract under condition of ATP depletion. Experiment like in D, except that the initial ATP concentration was 25 μM instead of 1 mM and apyrase (2 units) was added at time 0, to deplete ATP.

A brief, 2-minute incubation of blast-frozen MIN6 cells with a hypotonic, detergent-free buffer with ATP/Mg^2+^ was sufficient to extract proteasome complexes, as evidenced by Western blot analysis with antibodies specific to the representative 20S subunits α1–7, β5, β5_i_, the 19S subunits Rpt1 and Rpt5, and the 11S subunits α and β (Fig. 4A, lane 1). No major amounts of additional proteins were extracted by additional incubations with the same buffer (Fig. 4A, lanes 2–5) and cell pellet remaining after (p.a.) these extractions contained only a small fraction of the total proteasomes (Fig. 4A, lane 6). This fraction was extracted by additional incubation with 0.5% Triton X-100 (Fig. 4B, 20S β5, lane 6), a detergent that also extracted the ER protein Calnexin and the nuclear protein Sal1 (Fig. 4B, Calnexin, Sal1), but not the chromatin bound histone H3 (Fig. 4B, histone H3). Thus, the majority of the total cellular proteasomes was rapidly extracted with a hypotonic buffer without detergent and this fraction represented proteasomes that were cytosolic, or only loosely associated with the cytosolic side of the ER or nucleus.

To quantitate the amounts of the 20S cores and activators in the detergent-free extracts we performed quantitative Western blot analysis. The extracts were standardized by total protein content and analyzed in a range of protein concentrations (4, 2 and 1 μg), with equal loading confirmed with antibodies specific to GAPDH (Fig. 4C, GAPDH). Similar amounts of the 20S alpha subunits were detected in each extract (Fig. 4C, 20S α1–7, lanes 1–3, 4–6 and 7–9). The extracts had equal amounts of β5 subunit (Fig. 4C, 20S, β5, lanes 1–3, 4–6 and 7–9) and different amounts of β5_i_ subunit that was detectable only after exposure to IFNs (Fig. 4C, 20S, β5_i_, lanes 1–3, 4–6, and 7–9). Since all detectable alpha, β5, and β5_i_ subunits extracted under more harsh conditions were incorporated into the 20S cores (Fig. 3A), the equal amounts of constitutive subunits likely reflected equal amounts of the 20S core particles that differ in their β5_i_ content. Western blot analyses with antibodies specific to the 19S subunits Rpt4 and Rpt5 suggested a minor, less than 2-fold accumulation of the 19S in MIN6 cells exposed to IFNs (Fig. 4C, 19S, Rpt4 and Rpt5, lanes 1–3, 4–6 and 7–9), consistent with a similar increase in the 19S mRNAs levels (Fig. 1B). The 11S α and β proteins were approximately 2-fold more abundant after exposure to IFNβ and 4-fold more abundant after exposure to IFNγ compared to the amounts detected in untreated cells (Fig. 4C, 11S α and β, lanes 1–3, 4–6, 7–8). These findings showed that exposure to IFNs did not change the total levels of the 20S particles, but increased the levels of proteasomal activators.

Detergent-free MIN6 cell extracts verified to have equal levels of the 20S proteolytic cores were analyzed in peptidase assays for chymotrypsin-like activity with the Suc-LLVY-AMC reporter, which emits fluorescence after AMC release by proteolytic cleavage (Methods). This assay did not discriminate between the activities of β5 and β5_i_ subunits, instead scoring the total chymotrypsin-like activity of all mature 20S particles as long as they were associated with at least one activator. We first tested the proteolytic activity mediated by the ATP-dependent 19S activator. In assays with 1 mM ATP, all extracts had a catalytic rate of 0.1–0.15 fluorescence units (equivalent to an average of 15 pmol of liberated AMC) per minute that was stable for at least 20 minutes (Fig. 4D, -MG132). These results suggested that IFNs did not alter proteasomal activity facilitated by the ATP-dependent 19S activator. A similar conclusion was suggested by the finding that polyubiquitinated species, which are recruited to the proteasome in a manner dependent on the 19S activator, were degraded with similar rates during an incubation at 37°C (Fig. 4D, insert with Western blot). The proteasome inhibitor MG132 reduced the catalytic rate to 0.008 fluorescence units per minute (equivalent to 7% of the initial rate, or 1 pmol of liberated AMC; Fig. 4D, +MG132), verifying that the release of AMC was catalyzed by the proteasome.

To create conditions that would make the function of the 20S cores dependent on the ATP-independent 11S activator, we performed similar assays under condition of ATP depletion. Surprisingly, dilution of the initial extracts into buffer without ATP, which rapidly reduced ATP concentration from 1 mM to 25 μM, was sufficient to stimulate the catalytic rate by about 3-fold in all extracts (from 0.1 to 0.3 fluorescence units/min; compare Fig. 4E, time 0 with Figure 4D). This catalytic rate was gradually reduced upon incubation with apyrase, which depletes ATP, and stabilized within 10–20 minutes. The final catalytic rates typical of ATP depletion were approximately 2-fold higher in extracts from cells treated with IFNs compared to untreated cells (Fig. 4E, 20 minutes), and to rates measured at 1 mM ATP, which were similar in all extracts (Fig. 4E, 20 minutes, and Fig. 4D). These findings suggested that the 20S proteasomes were active in both the presence and absence of ATP. However, only under conditions of ATP depletion did the catalytic rates in IFN-treated cells exceed the catalytic rate in untreated cells in a manner that could be attributed to an accumulation of the 11S activator.

### Mouse islets have chronically elevated levels of the immune proteolytic subunits, but both mouse islets and MIN6 cells have chronically elevated levels of the 11S activator

To determine how well MIN6 cells recapitulate the IFNβ-mediated induction of immuno-proteasome function in primary β-cells, we also compared the extraction profiles and protein levels of the regular and inducible proteasomal subunits and activators in MIN6 cells and islets.

**Figure 5:**
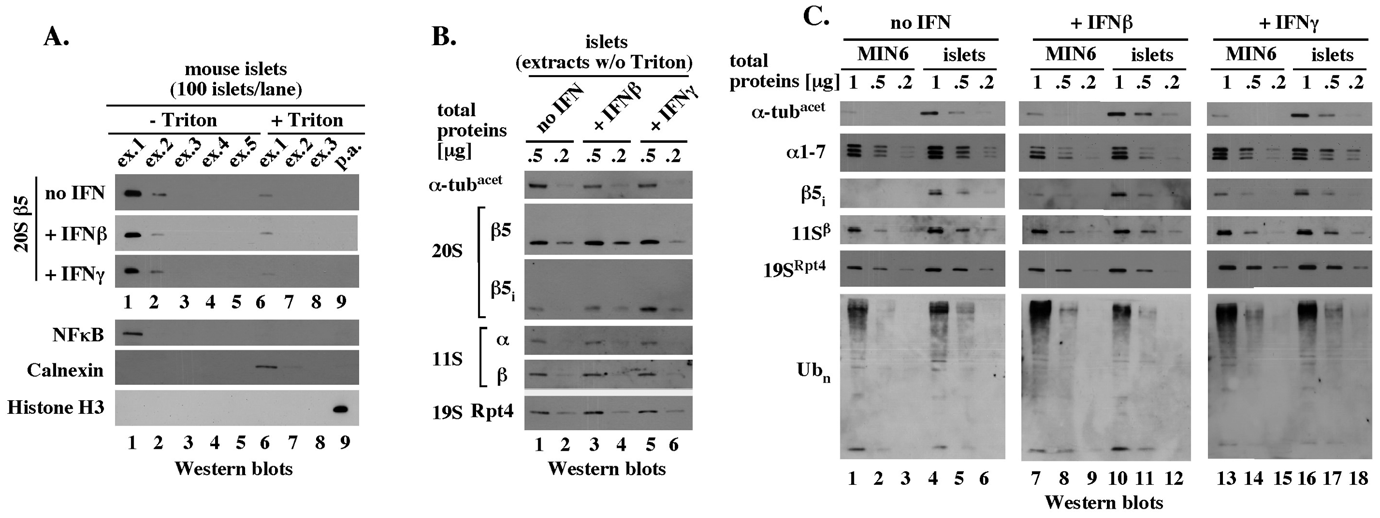
Mouse islets and MIN6 cells accumulate similar levels of the 20S, 19S, 11S and polyubiquitin chains. (A). Experiment like in Fig. 4B, except with mouse islets. (B). Experiment like in Fig. 4C, except with mouse islets. (C). Side-by-side Western blot analysis of the indicated, detergent-free extracts prepared from MIN6 cells and mouse islets was performed with the indicated antibodies, as described in Methods.

As in experiments with MIN6 cells, the majority of the representative 20S β5 subunit was extracted from mouse islets by a brief incubation with a hypotonic, detergent-free buffer, while Triton X-100 extracted the remaining 20S β5 (Fig. 5A, 20S β5, no IFN, lanes 1 and 6). The same extraction profiles were observed after treatment with IFNβ and IFNγ (Fig. 5A, 20S β5). All detergent-free extracts contained the cytosolic form of NFκB, but not the ER protein Calnexin, or the chromatin-associated histone H3 (Fig. 5A, and data not shown). Thus, regardless of IFN treatment, the majority of total proteasomes in mouse islets was cytosolic, or only loosely associated with the cytosolic side of the ER or nucleus.

In detergent-free extracts standardized by equal protein content determined by the Bradford assay and verified by equal levels of acetylated α-tubulin (Fig. 5B, α-tub^acet^), similar amounts of the constitutive 20S β5 subunit were detected regardless of the *in vitro* treatment with IFNs (Fig. 5B, 20S β5, lanes 1–2, 3–4 and 5–6). Side-by-side comparison of mouse islets and MIN6 cells verified similar amounts of the 20S alpha subunits (Fig. 5C, α1–7, compare lanes 1–3 and 4–6, 7–8 and 10–12, 13–15 and 16–18), suggesting that both cell types contained similar levels of the 20S particles. Comparison of MIN6 cells and mouse islets also revealed no major difference in the levels of the 19S activator or high molecular weight polyubiquitinated species, a large fraction of which typically represents proteasome substrates recruited by the 19S (Fig. 5C, 19S^Rpt4^ and Ub_n_).

However, while the inducible β5_i_ subunit accumulated in islets exposed *in vitro* to IFNβ or IFNγ, β5_i_ protein was also present in untreated islets in amounts only modestly lower than that observed after exposure to IFNβ (Fig. 5B, 20S β5_i_, compare lanes 1–2 with 3–4 and 5–6). MIN6 cells and mouse islets accumulated similar amounts of the β5_i_ subunit after exposure to IFNγ (Fig. 5C, β5_i_, lanes 13–15 and 16–18), suggesting that β5_i_ was expressed to a similar level in each cell type. However, in three separate preparations, islets consistently had an elevated initial level of β5_i_ protein (Fig. 5C, β5_i_, lanes 1–3 and 4–6; and data not shown). We made a similar observation about the 11S subunits α and β, which were present in similar amounts in isolated mouse islets regardless of whether they were untreated or exposed to IFNs *in vitro* (Fig. 5B, 11S α and β). In side-by-side comparison, the 11S subunits were expressed to similar maximal levels in MIN6 cells and mouse islets, but considerable accumulation of the 11S was observed even in untreated MIN6 cells (Fig. 5C, 11Sβ, lanes 1–3 and 4–6, 7–7 and 10–12, 13–15 and 16–18).

Overall, MIN6 cells and mouse islets accumulated similar levels of the 20S, 19S and 11S, and were comparable as models to study proteasome function and regulation. However, while untreated MIN6 cells did not express the inducible 20S subunits, these subunits were expressed at chronically elevated levels in untreated mouse islets. Furthermore, both MIN6 cells and mouse islets had chronically elevated levels of the 11S.

## Discussion

In this study, we tested how the immunoproteasome, a key protease implicated in antigen processing and type I diabetes onset, is controlled in pancreatic β-cells. Evidence was presented about 15 years ago that pancreatic β-cells express components of the immunoproteasome in response to IFNγ in a manner linked to a change in antigen presentation [35]. However, no study has analyzed the accumulation, assembly, and function of the immunoproteasome in pancreatic β-cells. Perhaps more importantly, our study is the first to implicate immunoproteasome in the early, IFNβ-mediated antiviral defenses of pancreatic β-cells that only recently emerged as a factor in the development of the diabetic state in patients and animal models [6]–[13].

Our data suggest that IFNγ and IFNβstimulate the expression of immune 20S and 11S genes in a fundamentally similar manner. In both cases, the accumulation of specific mRNAs was observed in similar time periods, with maximal accumulation at 12 hours and a decline by 24 hours, and was dependent primarily on the inducible IRF1 transcription factor. While IRF1 was known to facilitate the induction of the immunoproteasome and of the 11S genes by IFNγ [46]–[51], our study is the first to demonstrate that IRF1 plays the same role in the presence of IFNγ and IFNβ. This observation suggests that, at least in β-cells, a continuity of the coordinated expression of immunoproteasome and 11S genes could be ensured during the early and late stages of antiviral β-cell response via the involvement of the same transcription factor. A further insight into this control comes from the observation that IRF1 protein has a short half-life [49], suggesting that IRF1 levels closely match the levels of interferons. Since the turnover of the immunoproteasome is faster than the proteasome (T_1/2_ 21 vs 120 h) and independent of cytokines [52], [53], immunoproteasome levels could also closely mimic changes in IFNβ and IFNγ levels. The inability to regulate IRF1 [37] and/or ensure such a link could explain why IFNα induces only modest accumulation of the immunoproteasome and of the 11S activator [4] (data not shown).

How is the function and regulation of the early immunoproteasome in pancreatic β-cells different from the function and regulation of the classic, mature immunoproteasome? One major difference is suggested by the observation that the early 20S proteolytic cores contain stochastic combinations of regular and immune proteolytic sites. This type of arrangement has been also observed during the early steps of IFNγ signaling [23] (this work), but differs from the complete replacement of proteolytic subunits observed during long-term exposures to IFNΥ. How could the stochastic arrangement of proteolytic sites affect antigen processing? Complete replacement of proteolytic subunits is characteristic of the mature immunoproteasome, but the stochastic arrangement of proteolytic sites would likely be associated with a higher number of variations in protein cleavage and could more rapidly increase the probability with which unique immunogenic peptides are generated even from normally non-immunogenic and/or self-substrates. This model suggests that if such a change were sufficient for the early stimulation of the immune response against β-cells, it could also predispose to autoimmune responses under any conditions that cause even modest accumulation of the immune subunits. Our data suggest that such a modest accumulation could be more frequent than previously anticipated, as we find that the expression of immunoproteasome and 11S genes is controlled by IFNγ and IFNβ; that transcription factors other than IRF1 could be involved in the regulation by IFNβ; and that it is difficult to prepare mouse islets that completely lack the immune subunits without an extra *in vitro* treatment.

**Figure 6:**
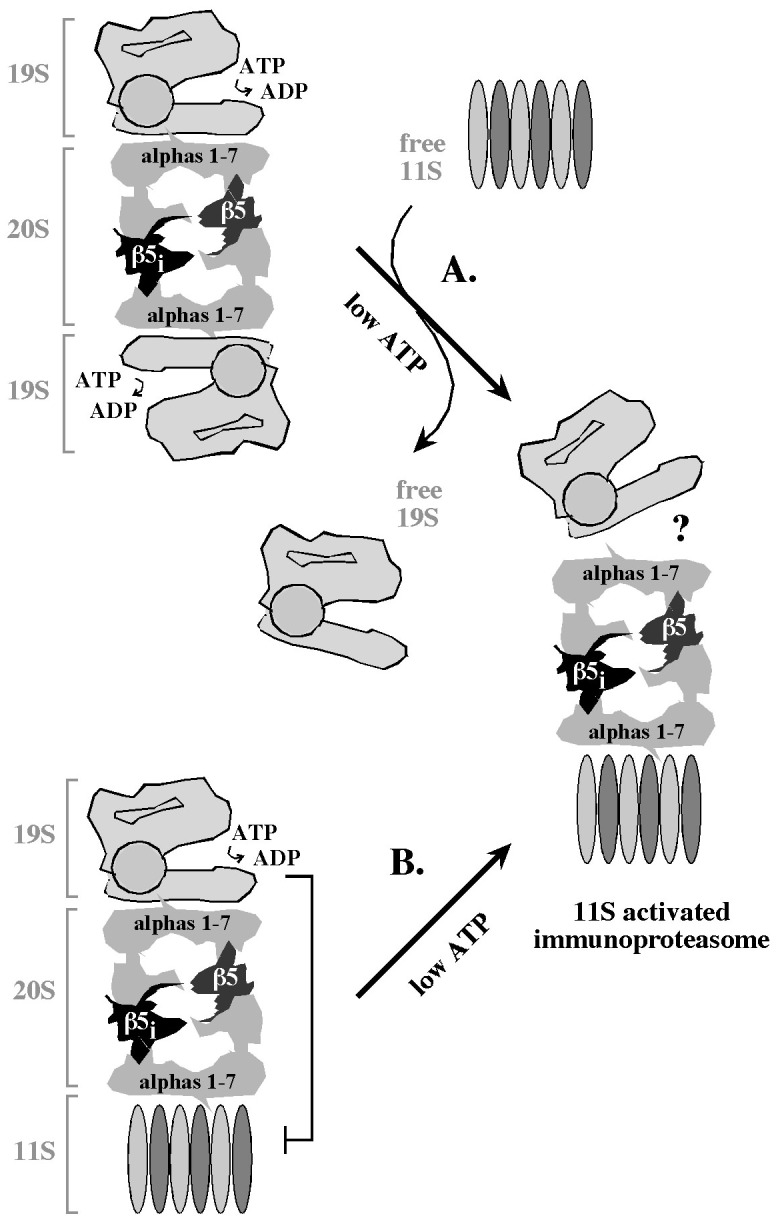
Summary model – the 11S activates the immunoproteasome in response to reduced ATP levels. In pancreatic β-cells exposed to IFNs, early immunoproteasome 20S cores have stochastic combinations of regular and immune proteolytic subunits, and coexist with the 19S and 11S activators. However, only under conditions of ATP depletion the proteolytic rates are stimulated in a manner consistent with the levels of the 11S activator. Two mechanisms could explain this observation. (A). The 20S cores could be saturated with the ATP-dependent 19S activators, and the 11S would bind the 20S cores only when at least one of the two 19S complexes dissociates in a manner stimulated by low ATP. (B). The 11S could be incorporated into hybrid 19S/20S/11S particles, but their proteolytic function would depend primarily on the 19S activator until ATP concentrations drop. Question mark emphasizes that it is unclear whether the final, 11S-activated immunoproteasome lacks the 19S activator. See text for details.

The second major finding regards the issue of early immunoproteasome regulation by the 19S and the 11S activators. We found that IFNβ or IFNγ stimulates the accumulation of the 11S activator in pancreatic β-cells. However, analysis of the proteolytic rates in cell extracts revealed that the accumulated 11S stimulates the proteolytic function of the immunoproteasome only under conditions of ATP depletion. A mechanism that restricts the function of the 11S activator in cells with high ATP levels could have a major regulatory significance, as it could suppress the generation of immunogenic peptides even if cells accumulated the immunoproteasome and the 11S activator, and could rapidly activate antigen processing but only when ATP levels drop. What could be the molecular basis of such a regulatory phenomenon? A reduction in ATP concentration could be necessary to activate 11S participation when the 20S cores are saturated with the ATP-dependent 19S activators and need to be freed from at least one of the two 19S complexes prior to the 11S binding (Fig. 6A). In support of this possibility, we observed a modest accumulation of the 19S components during exposure to IFNs that could restrict premature access of the 11S activator to the 20S particles. Another possibility is that high ATP levels restrict the function of the ATP-independent 11S activator in the context of hybrid 11S/20S/19S particles (Fig. 6B). This model predicts the existence of an allosteric mechanism that coordinates the function of the two activators within a single 11S/20S/19S particle, which would be a new concept.

Regardless of the 11S activation mechanism, its dependence on ATP depletion suggests that the activation of 11S could be also associated with a change in the pool of substrates. Indeed, the 11S recruits substrates in a manner independent of polyubiquitination, which would likely be limited at low ATP levels, thereby further restricting the role of the 19S activator. The only substrates known to be recruited specifically via the 11S activator are cyclin-dependent kinase inhibitors p21^Cip^, p16^INK4A^, and p19^ARF^ [32], but it cannot be excluded that the 11S facilitates degradation of additional proteins, including proteins damaged by reactive oxygen and nitrogen species that are robustly produced in cells exposed to IFNs. Apart from its role in antigen generation, rapid proteolysis of damaged proteins could also play a role in promoting β-cell survival, similarly to the protective role of the immunoproteasome during IFNγ-induced oxidative stress [54].

While these possibilities are intriguing, the question remains, what are the variations in ATP concentrations and immunoproteasome function in pancreatic β-cells *in vivo*? In general, insulin secretory function is tightly linked to changes in ATP concentrations, but little is known about how ATP concentrations are regulated during viral infection and/or antiviral responses. In response to inflammatory cytokines, such as IL-1 and combinations of IL-1 and IFNs, the total cellular ATP levels are decreased by 5-fold and this change depends on enhanced expression of the inducible isoform of nitric oxide synthase (iNOS) and production of nitric oxide [55], [56]. Since iNOS expression, nitric oxide production, and the generation of inflammatory cytokines such as IL-1 are stimulated by synthetic dsRNA that is used to mimic virus infections [57], it is interesting to speculate that a reduction in ATP levels may link the effects of iNOS expression and nitric oxide production to immunoproteasome activation by the 11S, and may result in the generation of altered peptides that may be antigenic. Future studies on these issues will be possible thanks to the emerging new technologies for monitoring ATP levels in intact cells. For example, the recent development of a fluorescent probe that can detect changes in ATP concentration ranging from 7.4 μM to 3.3 mM revealed that ATP levels in the mitochondrial matrix of HeLa and NIH 3T3 cells are significantly lower than those in the cytoplasm and nucleus, and that ATP levels are synergistically regulated by rapid changes in glycolysis and oxidative phosphorylation [58]. The possibility that proteasome function is affected by these changes is supported by the observation that the catalytic activity of proteasome is dramatically activated at approximately 25 μM ATP in pancreatic β-cell extracts (this work) and during myocardial injury in cold ischemia [59]. The emerging new technologies for monitoring proteasome function *in vivo* [60] may enable future verification of these findings in intact cells.
