# Expression of Concern: Reduction in ATP Levels Triggers Immunoproteasome Activation by the 11S (PA28) Regulator during Early Antiviral Response Mediated by IFNβ in Mouse Pancreatic β-Cells

**DOI:** 10.1371/journal.pone.0229516

**Published:** 2020-02-18

**Authors:** The PLOS ONE Editors

Concerns have been raised about Figs 2 and 3 of this article [1]. Specifically:

- In Fig 2B, there appears to be vertical discontinuity between lanes 1 and 2 in the β5_i_ panel.
- In Fig 3A, similarities were noted between areas in lanes 8–14 and lanes 15–19 of the α1–7 (IFNβ, 24h) blot, and between areas in lanes 9–13 and areas in lanes 14–17 of the α1–7 (no IFN, 24h) blot.
- In Fig 3C, there appear to be vertical discontinuities between lanes in the α1–7 (after lanes 1, 2, 5, 6), β5^C^ (after lanes 1, 2, 3, 7), and β5_i_^M^ (after lane 7) panels.

For Figs 2B and 3C, the corresponding author noted that each figure panel in question was prepared using data obtained from the same blot and exposure, and that some lanes from the original blot were spliced out when preparing these figures. Incorrect image data were included in the IgG control (“ctr IgG”) lanes of the α1–7 and β5^C^ panels in published Fig 3C. These issues have been addressed in the updated figures provided with this notice. Original blots supporting Figs 2B and 3C are provided in Supporting Information S1–S3 Files, with the exception of the image supporting lanes 1–4 of the β5_i_^M^ blot in Fig 3C which is no longer available.

For Fig 3A, the corresponding author explained that the same blots were probed with anti-β5 or anti-β5_i_ antibody, then stripped and reprobed with anti-α1–7. β5 signal was still visible in lanes 14,15, and 17 after the blots were stripped and reprobed, and this residual signal was obscured in the published α1–7 figure panels. In addition, the original figure did not include the matched α1–7 data for the β5_i_ experiment. These issues are addressed in the updated figure and the underlying blot images are in S2 File. In the updated figure, the residual β5 or β5_i_ signal is indicated with asterisks. The author commented that they have not observed the α1–7 antibodies to cross-react with β5 or β5_i_.

The raw data underlying all other results reported in the article are available upon request from the corresponding author.

Updated Figs 2 and 3 and their respective figure captions are provided here. A member of *PLOS ONE*’s Editorial Board advised that the updated figures support the results and conclusions reported in the original article. However, the *PLOS ONE* Editors issue this Expression of Concern due to concerns about how the above western blot data were reported in the original article.

**Fig 2:**
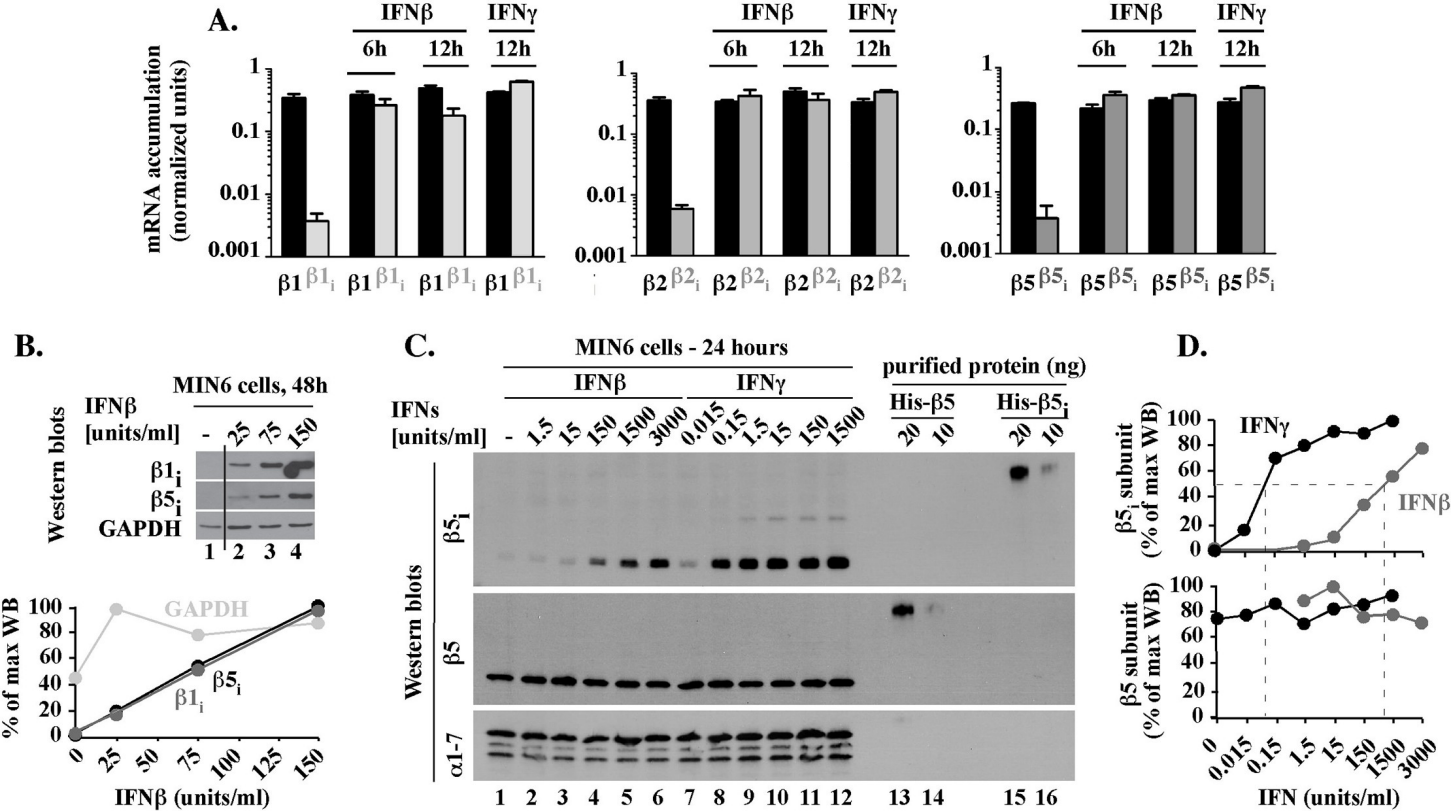
In MIN6 cells exposed to IFNβ, inducible and regular 20S mRNAs and proteins are expressed concurrently and in similar levels. (A). Comparison of inducible and regular 20S mRNA levels. mRNA levels of regular (black, β1, β2, β5) or immune (gray, β1_i_, β2_i_, β5_i_) subunits in MIN6 cells treated with 150 units/ml IFNβ or IFNγ for 6 or 12 hours were tested by qPCR, procedure B. Results are the mean ± S.E.M. of three experiments. (B). Accumulation of inducible 20S β1_i_ and β5_i_ proteins (revised). Whole cell extracts from MIN6 cells treated for 48 hours with various concentrations of IFNβ were analyzed by western blot (WB) with antibodies specific to β5_i_ (~20 kDa) followed by stripping and re-probing of the same membrane with antibodies specific to GAPDH (~38 kDa) that served as both a loading control and a verification that the β5i-specific signal was removed. The same membrane was then stripped again and re-probed with antibodies specific to subunit β1_i_ (~23 kDa). The black vertical line indicates the removal of three lines with IFNα treated cell samples analyzed in the same experiment. These lanes, which are now shown in the Supplement to Fig 2B, were removed solely because the effects of IFNα were not a subject of the report. In addition, the original experiment included a side-by-side reference of IFNγ (150 U/ml, Supplement to Fig 2B), to verify that the results were consistent with the more extensive analysis shown in panel C. Quantitation of WB data is shown as a percentage of the maximal accumulation for each analyzed protein. (C). Comparison of β5 and β5_i_ protein levels. MIN6 cells exposed for 24 hours to the indicated concentrations of IFNβ or IFNγ (lanes 1–12) were analyzed by western blot next to 10 and 20 ng of mouse His-β5 and His-β5_i_ proteins expressed in, and purified from, *E*. *coli* (lanes 13–16). Levels of the 20S alpha subunits (α1–7 WB) are shown as loading control. (D). Quantitation of WB data presented in C. Protein levels represent a percentage of the maximal accumulation for each protein.

**Fig 3:**
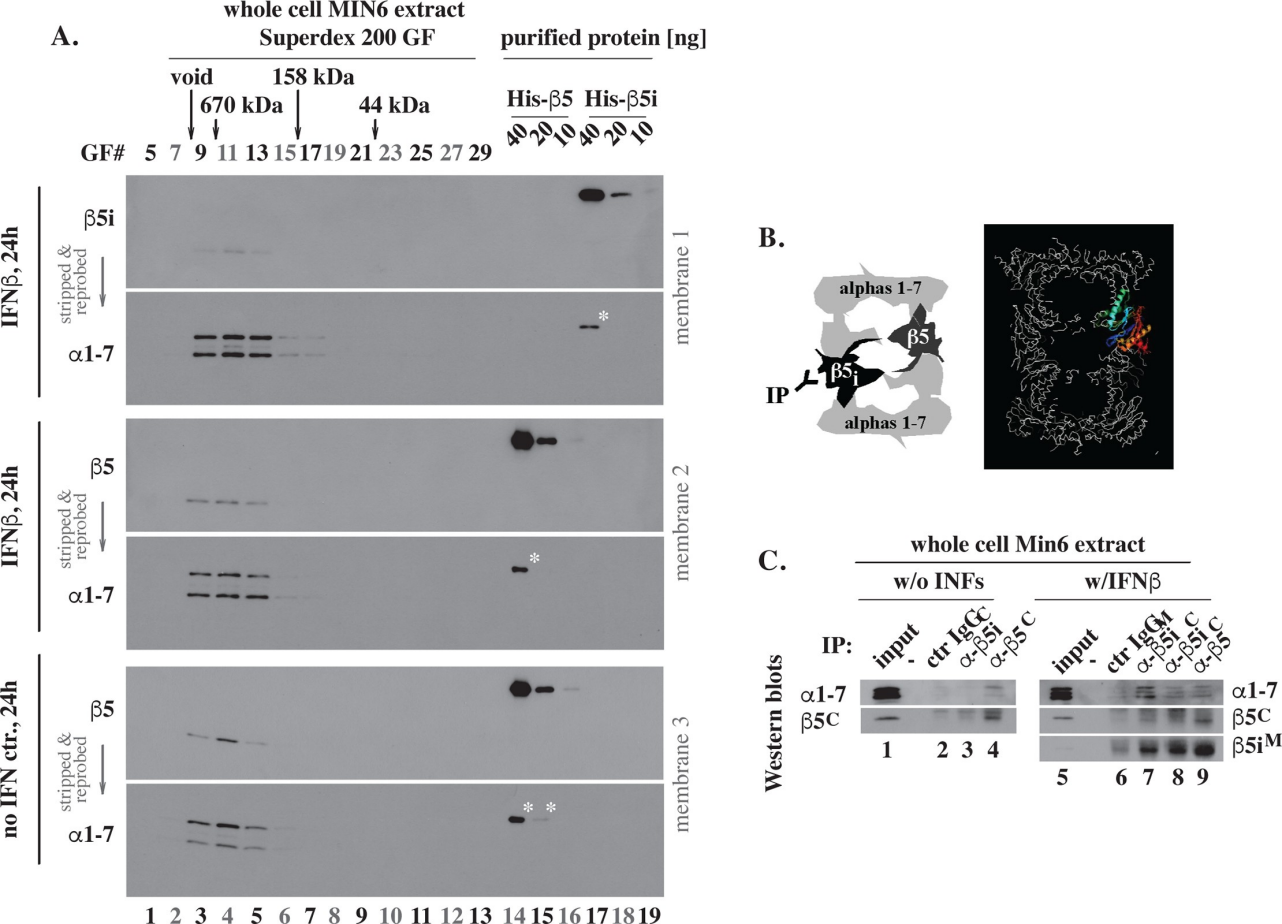
After 24 hours of treatment with IFNβ, inducible and regular subunits co-exist in the 20S particles. (A). Size exclusion chromatography (revised). Size exclusion chromatography of extracts prepared from IFNβ treated (IFNβ, 24h) and untreated (no IFN ctr., 24h) MIN6 cells was performed as described in Methods and the indicated gel filtration fractions (GF# 5–29, lanes 1–13) were analyzed by western blot on three separate membranes, as indicated. Each membrane was first probed for β5_i_ or β5, as indicated, followed by stripping and re-probing for alphas (α1–7). The stripping and re-probing approach generated reliable outcomes, as evidenced by the differences in patterns (a single band of β5_i_ or β5 vs multiple bands of α1–7), apparent molecular weights (20kDa vs 30kDa, respectively), and animal hosts used to generate the antibodies (rabbit vs mouse, respectively). In addition, the experiment included a side-by-side reference of mouse His-β5 and His-β5_i_ proteins expressed in, and purified from, bacteria (40, 20 and 10 ng each, lanes 14–19). This reference was included to emphasize two additional points. First, western blot detection was selective even with 40 ng of purified β5 and β5_i_ proteins (WB for β5 or β5_i_, lanes 14 and 17), while the intensities observed in MIN6 cell extracts (WB for β5 or β5_i_, lanes 3–5) were similar to 10 ng (WB for β5 or β5_i_, lanes 16 and 19). Second, signals associated with 10 ng of β5 or β5_i_ proteins were no longer detectable after stripping and re-probing for alphas (WB for α1–7, lanes 16 and 19), thereby providing yet another verification that the stripping and re-probing approach did not interfere with the detection of alpha subunits present in MIN6 cell extracts (WB for α1–7, lanes 3–5). Asterisks mark the previously obscured left-over amounts of rabbit antibodies specific to β5 and β5_i_ that were still detectable in lanes with the highest amount (40 ng) of purified proteins after stripping and re-probing with mouse antibodies specific to α1–7. Supplement to Fig 3A shows all the original western blots. (B). Models with β5 and β5_i_ subunits in a single 20S particle (left), and with 20S structure in which one of the two β5 subunits is marked in color, with C-terminus in red (right). (C). Immuno-precipitation (revised). MIN6 extracts analyzed by in panel A were subjected to immuno-precipitation (IP) using antibodies indicated on the top followed by western blot analysis as indicated on the sides. The published panel C was assembled from individual cuts, which were labeled correctly except for misplaced control images in lanes 2 and 6, and unmarked removal of one line with no samples loaded. The revised panel C now includes intact images copied from the original X-ray films stored in laboratory records (Supplement to Fig 3C). Only bands with the approximate molecular weights of mature proteins visible in the inputs are shown. The upper bands visible in the original data (Supplement to Fig 3C) represent heavy chains of the IgG used in the IPs. Multiple experiments and exposure times could be presented upon request, but the all-negative X-ray film with the IFN(-) β5_i_^M^ panel in lanes 1–4 was not saved and is no longer shown.

The authors apologize for the errors in the published article.

## Supporting information

S1 File Original blots supporting Fig 1B. (TIFF)

S2 File Original blots supporting Fig 2A. (TIFF)

S3 File Original blots supporting Fig 2C. (TIFF)
