# Supplementary figures and images for: Expression of Concern: Reduction in ATP Levels Triggers Immunoproteasome Activation by the 11S (PA28) Regulator during Early Antiviral Response Mediated by IFNβ in Mouse Pancreatic β-Cells
Source: PLoS One. 2020 Feb 18;15(2):e0229516. doi: 10.1371/journal.pone.0229516 (PMC7028283; doi:10.1371/journal.pone.0229516)

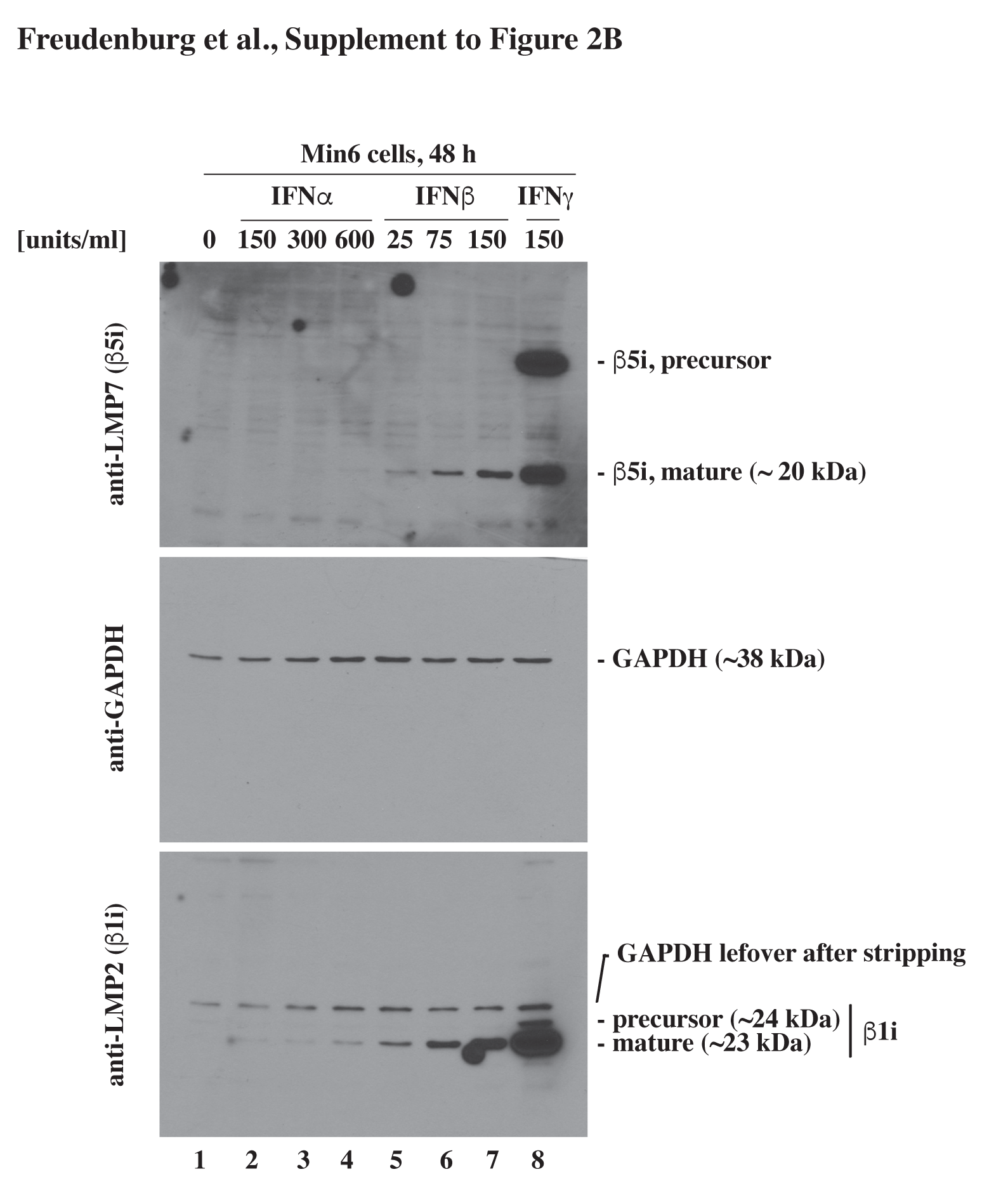

Supplement: S1 File — (TIFF) [file pone.0229516.s001.tiff]

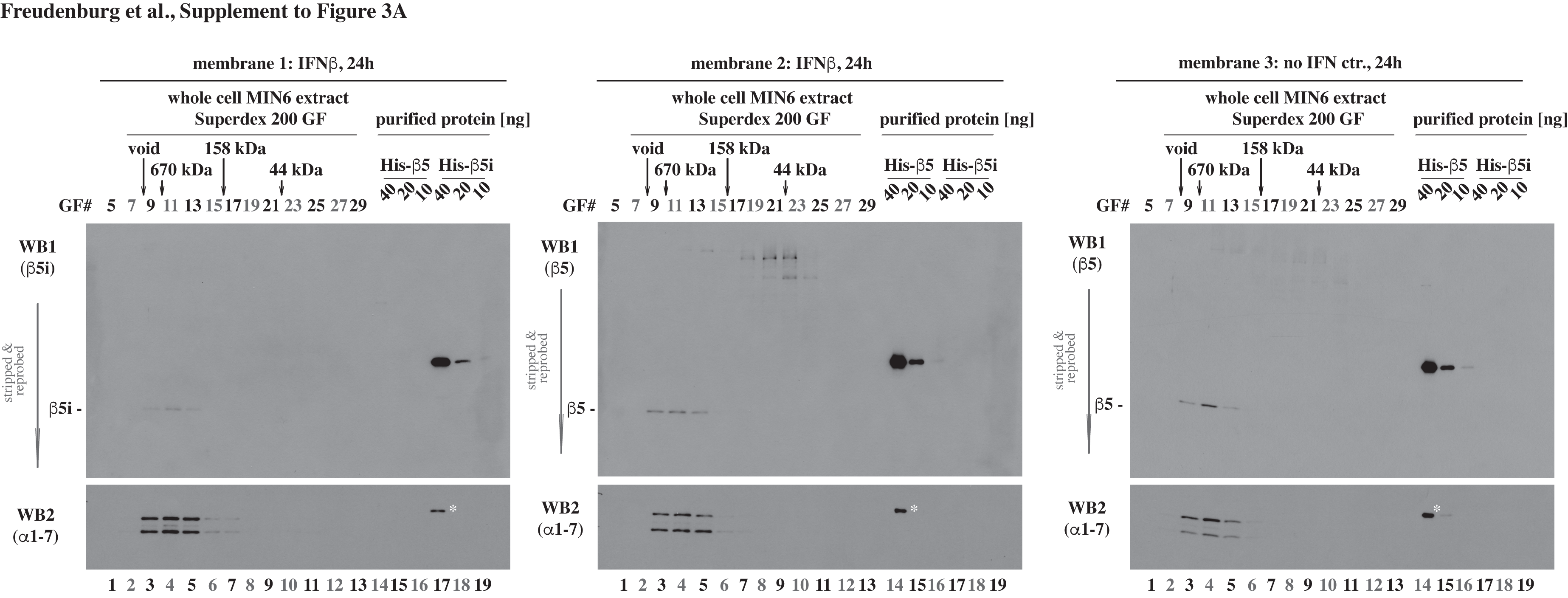

Supplement: S2 File — (TIFF) [file pone.0229516.s002.tiff]

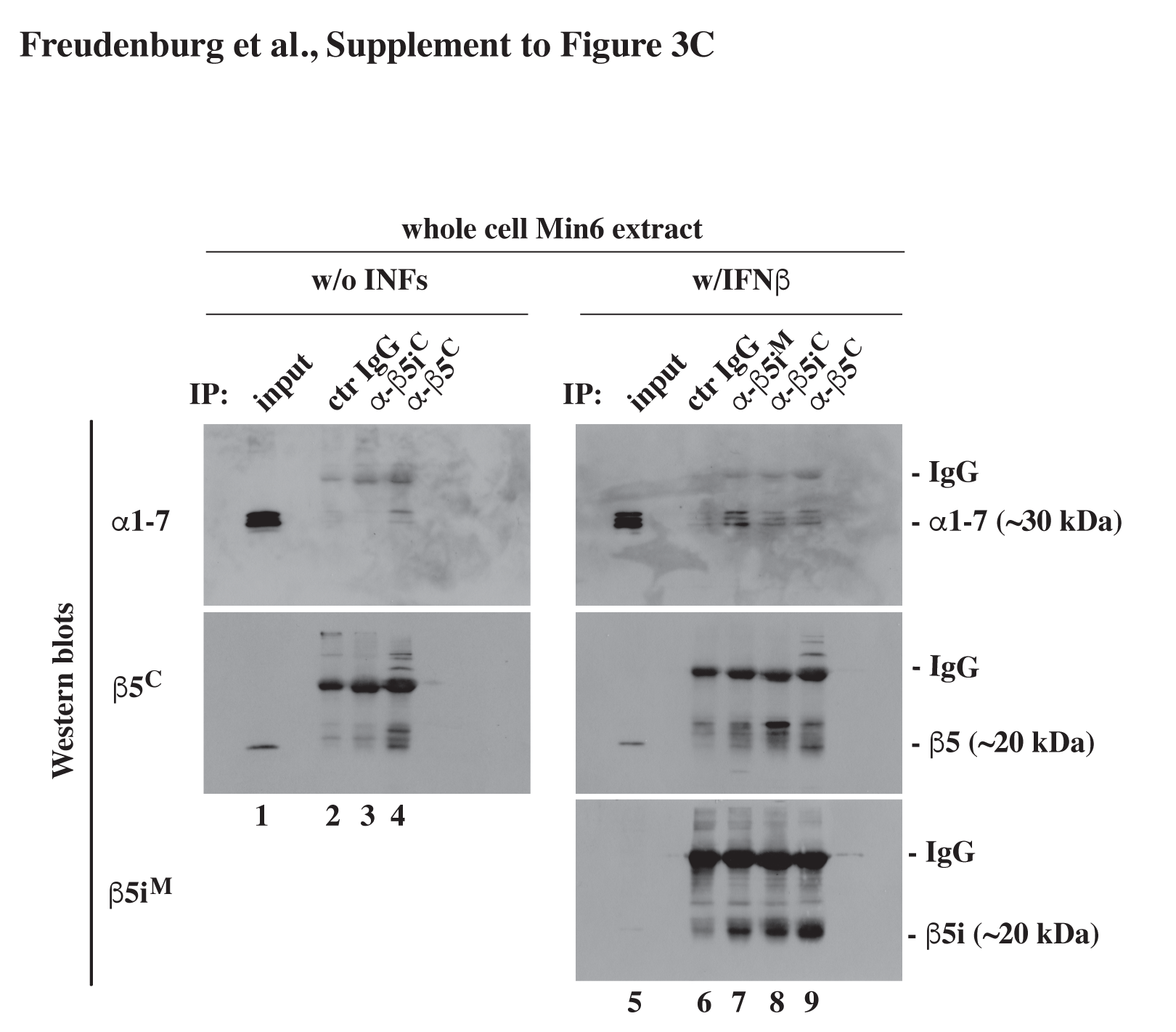

Supplement: S3 File — (TIFF) [file pone.0229516.s003.tiff]
